# Fertility problems in men carrying a translocation involved in breakpoints on chromosome 17p13: A retrospective, observational study

**DOI:** 10.1097/MD.0000000000032216

**Published:** 2022-12-09

**Authors:** Ranwei Li

**Affiliations:** a Department of Urology, the First Hospital of Tsinghua University, Beijing, China; b School of Clinical Medicine, Tsinghua University, Beijing, China.

**Keywords:** breakpoint, genetic counseling, male infertility, reciprocal chromosomal translocation

## Abstract

Male infertility is a multifactorial reproductive disorder. The effect of genetic factors on male infertility has been the focus of research. Although a variety of genetic techniques are applied to male infertility in clinical practice, karyotype analysis remains a powerful and inexpensive technology. Reciprocal chromosomal translocation (RCT) is closely related to male infertility, but the clinical phenotypes of RCT carriers are varied, and the underlying pathological mechanism is unclear. Some studies suggest that RCT breakpoints disrupt the structure and function of important genes responsible for spermatogenesis. Several breakpoints of chromosome 17 are related to important genes, which can lead to spermatogenic failure. This study aimed to identify the clinical features of 3 men with translocation karyotypes involving breakpoints on chromosome 17p13. Semen analysis and cytogenetic analysis were performed with informed consent. Gene ontology analysis was performed for 60 pathogenic genes on chromosome band 17p13. Cytogenetic analysis showed that the karyotypes were 46, XY, t(6;17) (p21;p13), 46,XY,t(10;17)(q11.2;p13), and 46, XY, t(17;20) (p13;q13), respectively. Relevant studies and genes on breakpoints on chromosome 17p13 were searched for using PubMed. Fourteen reported cases of the same karyotype were reviewed. The results suggest that although chromosome 17 is closely related to spermatogenic failure, the clinical phenotypes of RCT carriers with involvement of 17p13 breakpoints are varied. The important genes involved in the breakpoint were analyzed. The results of molecular functions suggested that these targets genes on chromosome band 17p13 were mostly involved in microfilament motor activity, ATPase activity. These results suggested that the translocation chromosome and breakpoint analysis should be considered in the clinical assessment of the patients. Physicians should be aware of these in genetic counseling. These breakpoints and the function of related genes require further study.

## 1. Introduction

Male infertility accounts for approximately 50% of infertile couples, and it is a multifactorial reproductive disorder.^[1]^ The effect of genetic factors on male infertility has been the focus of research.^[2]^ Whole-exon sequencing has been applied in the clinical practice of nonobstructive azoospermia.^[3]^ Because sequencing technology cannot detect balanced chromosome rearrangements, chromosome analysis remains a powerful and inexpensive technology, and remains helpful for physicians and infertile patients.^[4]^ Reciprocal chromosomal translocation (RCT) is 1 of the most common types of human chromosomal abnormalities, and is closely related to male infertility.^[5]^ In clinical practice, male RCT carriers may show azoospermia, oligozoospermia, asthenozoospermia, or teratozoospermia, or their partners may increase the risk of recurrent spontaneous abortion.^[6,7]^ In addition, there are also RCT carriers who have a normal birth without any abnormal clinical manifestations. Therefore, the incidence of reciprocal translocation is often underestimated.^[8]^

The underlying pathological mechanism of RCT affecting male fertility is not completely clear. However, RCT breakpoints disrupt the structure and function of important genes responsible for spermatogenesis and lead to male infertility.^[9–11]^ Multiple RCT breakpoints have been found to be associated with male infertility.^[5,12–14]^ Among 75 spermatogenic (SPGF) failure phenotypes, 8 related genes have been located on chromosome 17 (https://www.ncbi.nlm.nih.gov/omim/). Hence, the breakpoints of chromosome 17 may be important for male fertility. However, the relationship between these translocation breakpoints and male infertility needs to be further examined in clinical practice.

This study aimed to identify the clinical features of 3 men with translocation karyotypes involving breakpoints on chromosome 17p13, and to examine the relationship between breakpoints and male infertility.

## 2. Methods

### 2.1. Study design and settings

This study was an observational, retrospective study conducted at the First Hospital of Tsinghua University. This study was approved by the Ethics Committee of the First Hospital of Tsinghua University. The need for informed consent was waived because of the retrospective design of this study.

### 2.2. Patients

The subjects of this study were 3 male carriers of RCTs. All patients visited the hospital to consult a doctor because their spouses had not given birth even after many years of marriage. After the physician asked about their medical history, the patients received semen analysis and cytogenetic analysis with informed consent. At the same time, relevant examinations of their spouses were recorded.

### 2.3. Semen analysis

Each patient was instructed to abstain from sex for 3 to 7 days, and semen samples were collected in a sterile container by masturbation. Semen parameters were detected using a computer-aided semen analysis system (Shanghai Beion Medical Technology Co., Ltd., Shanghai, China). Semen analysis was performed by 2 professional technicians according to the method recommended by the World Health Organization guidelines. Semen analysis consisted of sperm concentrations, sperm motility, sperm morphology, and progressive motility of sperm. All patients underwent semen analysis more than twice. No sperm in semen was diagnosed as azoospermia. Oligozoospermia was diagnosed when sperm concentrations were lower than 15 × 10^6^/ml. Sperm progressive motility < 32% were diagnosed as asthenospermia.

### 2.4. Cytogenetic analysis

Peripheral blood (2mL) from each subject was collected in sterile tubes containing heparin anticoagulant. Lymphocytes were cultured in 5-mL commercial culture medium (including 2% phytohemagglutinin, Yishengjun; Guangzhou Baidi Biotech, Guangzhou, China) for 72 hours at 37°C. Cultured lymphocytes were added to 0.1mL colcemid for 30minutes, and the lymphocytes were harvested. G-banding was then performed using standard procedures. Chromosome 400-band level and twenty metaphase cells were analyzed, and the karyotypes were described according to the International System for Human Cytogenetic Nomenclature (ISCN 2020).

### 2.5. Literature review

To examine the relationship between 17p13 breakpoints and clinical phenotypes, related genes on some breakpoints found in this study (6p21, 10q11.2, 20q13, and 17p13) were searched for using (online mendelian inheritance in man; https://www.ncbi.nlm.nih.gov/omim). Relevant studies involving chromosome17p13 translocation were searched for in PubMed using the following terms: chromosome translocation/male infertility. Cases with breakpoints on chromosome 17p13 were collected. Age ranges of literature are from 1980 to 2022.

### 2.6. GO analysis

Starting from the distal of the short arm of chromosome 17, 15Mb regions were analyzed, including regions 10.8Mb on chromosome band 17p13 and regions 4.2Mb downstream. Through DECIPHER （https://www.deciphergenomics.org/）search, 60 pathogenic genes were found, among which fertility related genes were identified. Meanwhile, gene ontology analysis (GO analysis) (https://david.ncifcrf.gov/home.jsp) was performed for these 60 pathogenic genes.

## 3. Results

**Figure 1.**
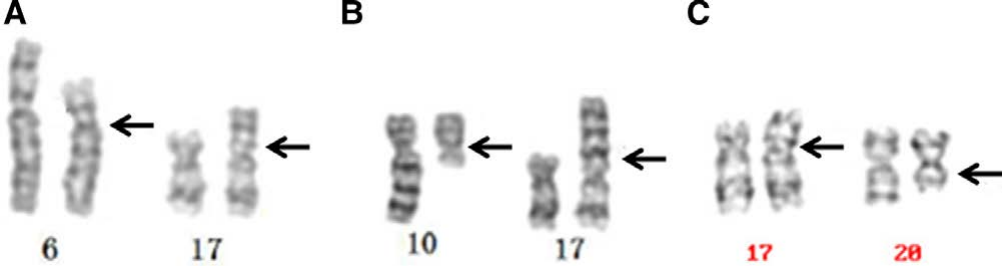
G-banding karyotypes of the 3 patients in this study.

Case 1 was a male patient aged 30 years with a normal phenotype and his spouse had not given birth in the 3 years after marriage. Case 2 was a 28-year-old man with a normal phenotype, and his wife had 3 consecutive spontaneous abortions. Case 3 was a 29-year-old man with a normal phenotype. Semen analysis results showed that case 1 had azoospermia, semen parameters of case 2 were within the normal reference value range, and case 3 had oligoasthenospermia. Cytogenetic analysis showed that the karyotypes of the 3 patients were 46, XY, t(6;17) (p21;p13) (Fig. 1a), 46, XY, t(10;17) (q11.2;p13) (Fig. 1b), and 46, XY, t(17;20) (p13;q13) (Fig. 1c). No abnormal changes were observed in routine clinical or genetic examinations of their spouses.

These RCT cases had 4 translocation breakpoints, all of which involved a breakpoint on chromosome 17p13. Relevant genes at these breakpoints were reviewed to analyze the relationship between these breakpoints and the clinical phenotype (Table 1). Seven important genes related to male infertility were found. The solute carrier family 26, member 8*, dynein, axonemal, heavy chain 8,* and muts homolog 5 (*MSH5*) genes are located on chromosome 6p21. The *SYCP2*gene, which is located on 20q13, is closely related to severe oligozoospermia. The *dynein, axonemal, heavy chain 2* (*DNAH2), zinc finger mynd-containing protein 15 (ZMYND15),* and *spermatid maturation protein 1* (*SPEM1*) genes are located on chromosome 17p13.

**Table 1:** Genes and their functions associated with translocation breakpoints in this study.

Breakpoint	Gene	Full name of gene	Loci	Function
6p21	*SLC26A8* (608480)	Solute carrier family 26, member 8	6p21.31	Asthenozoospermia
	*DNAH8* (603337)	Dynein, axonemal, heavy chain 8	6p21.2	Asthenoteratozoospermia
	*MSH5* (603382)	Muts homolog 5	6p21.33	Nonobstructive azoospermia
10q11.2	N/A	N/A	N/A	N/A
20q13	*SYCP2* (604105)	Synaptonemal complex protein 2	22q13.33	Severe oligozoospermia
17p13	*DNAH2* (619094)	Dynein, axonemal, heavy chain 2	17p13.1	Severe teratozoospermia
	*ZMYND15* (614312)	Zinc finger mynd-containing protein 15	17p13.2	Azoospermia
	*SPEM1* (615116)	Spermatid maturation protein 1	17p13.1	Playing a function in late stages of spermatid maturation

DNAH2 = dynein, axonemal, heavy chain 2, DNAH8 = dynein, axonemal, heavy chain 8, MSH5 = muts homolog 5, SLC26A8 = solute carrier family 26, member 8, SPEM1 = spermatid maturation protein 1.

To further examine the relationship between breakpoints on chromosome 17p13 and problems of fertility, 14 carriers were identified by performing a literature search. Including the 3 cases in this study, the clinical features of 17 RCT cases were collected and are shown in Table 2. The clinical fertility problems of these male carriers were varied. Of the 17 cases, the most common manifestation was that the spouses of these carriers had recurrent spontaneous abortions. In addition, there were 2 cases of oligozoospermia, 1 case of oligoasthenospermia, 1 case of azoospermia, and 1 case of normospermia.

**Table 2:** Clinical features of carriers of reciprocal chromosomal translocation involving chromosome 17p13 breakpoints.

Case	Karyotype	Seminal parameters	Reproductive history of the couple	Reference
1	t(1;17) (q21;p13)	N/A	Preimplantation genetic test	MM et al ^[25]^
2	t(1;17) (q21.3;p13.3)	N/A	Recurrent miscarriage	Pundir et al ^[27]^
3	t(1;17) (q44;p13.2)	N/A	Have abnormal child carrying an imbalance	Manvelyan et al ^[12]^
4	t(2;17) (q35;p13)	Normozoospermia	Abortion	Jenderny ^[28]^
5	t(4;17) (q21;p13)	Normozoospermia	Habitual miscarriage	Li et al ^[29]^
6	t(5;17) (q31;p13)	Normozoospermia	Infertility	Anton et al ^[23]^
7	t(6;17) (p21;p13)	N/A	Multiple abortion history	Davis et al ^[30]^
8	t(7;17) (p22;p13.2)	N/A	Recurrent miscarriages	Pasińska et al ^[31]^
9	t(8;17) (q24.1;p13.1)	N/A	Recurrent pregnancy loss	Stephenson et al ^[32]^
10	t(9;17) (q22.1;p13.1)	N/A	Recurrent spontaneous abortion	Ghazaey et al ^[33]^
11	t(11;17) (p15.5;p13.3)	N/A	Recurrent miscarriages	Joyce et al ^[34]^
12	t(13;17) (q12.3;p13.3)	Oligozoospermia	Infertility	Matsuda et al ^[35]^
13	t(17;19) (p13;p13)	Oligozoospermia	Assisted reproductive technology	Zhang et al ^[36]^
14	t(17;19) (p13.3q13.33)	Normozoospermia	Have abnormal child carrying an imbalance	Alvarado et al ^[24]^
15	t(6;17) (p21;p13)	Azoospermia	Infertility	This study
16	t(10;17) (q11.2;p13)	Normozoospermia	Recurrent spontaneous abortion	This study
17	t(17;20) (p13;q13)	Oligoasthenospermia	Infertility	This study

**Figure 2.**
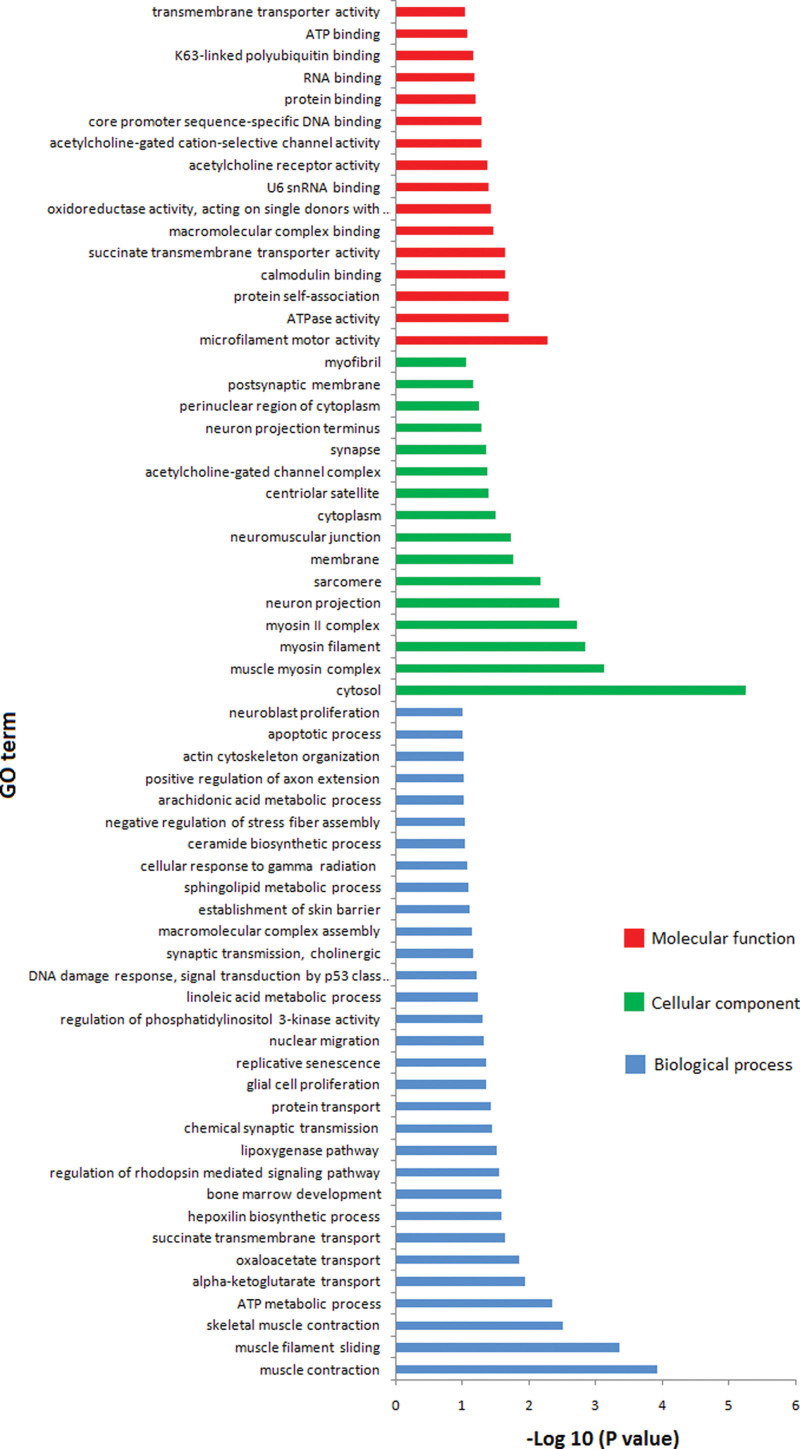
GO analysis results of genes located on chromosome 17p13. Abscissa represents–log (*P* value), and ordinate represents GO terms. Red represented molecular function; Green represented cellular component; Blue represented biological process. GO analysis = gene ontology analysis.

GO analysis showed that the target genes on chromosome 17p13 are strongly involved in cellular component, biological processes, and molecular function. The results of cellular component suggested that these target genes mainly localized at cytosol. The results of molecular functions suggested that these target genes were mostly involved in microfilament motor activity, ATPase activity. The results of biological processes suggested that these target genes are involved in muscle contract, muscle filament slicing, skeleton muscle contract, ATP metabolic process, et cetera (Fig. 2).

## 4. Discussion

Reciprocal translocation is a causative factor of male infertility.^[15]^ Translocation breakpoints may be responsible for the failure of spermatogenesis, depending on the chromosomes involved.^[16]^ In RCT carriers, potential gene disruptions or dysregulation maybe the contributing mechanisms that underlie male infertility.^[17]^ In clinical practice, semen parameters may be abnormal or within the normal range for different RCT carriers.^[18]^ Some RCT carriers have partners that give birth to children without any abnormal performance, while in others, their partners have repeated spontaneous abortions.^[8]^ Cases 1 and 3 in this study showed azoospermia and oligoasthenospermia respectively, and case 2 showed that his wife had recurrent spontaneous abortion. Therefore, genetic counseling for these RCT carriers is still a challenge.

Spermatogenic failure is the most severe form of male infertility.^[19]^ There are many genes closely related to azoospermia in online mendelian inheritance in man, and 8 of these genes are located on chromosome 17. The *ZMYND15* gene (614312), which located at chromosome 17p13.2, is closely associated with azoospermia.^[20]^ The *DNAH2* gene (603333) has been mapped to chromosome 17p13.1, and is related to severe teratozoospermia.^[21]^ The *SPEM1* gene to chromosome 17p13.1 is predicted to function in late stages of spermatid maturation.^[22]^ The 3 RCT carriers in this study included 4 breakpoints: 6p21, 10q11.2, 17p13, and 20q13. The *SLC26A8, DNAH8,* and *MSH5* genes are located on chromosome 6p21. The *DNAH2, ZMYND15,* and *SPEM1* genes are located on chromosome 17p13. By analyzing the relationship between these genes and phenotype, we speculated that case 1 showed azoospermia, which may have been related to the disruption of the *MSH5* or/and *ZMYND15* gene. Meanwhile, the *SYCP2* gene, which located on 20q13, is closely related to severe oligozoospermia. We speculated that the phenotype of case 3 may be related to this gene. The repeated spontaneous abortion of the wife in case 2 may be related to unbalanced gametes.

In order to further explore the clinical manifestations of RCT carriers with breakpoint 17p13, 17 cases were collected. Of the 17 previously reported RCT cases, 9 were diagnosed owing to repeated spontaneous abortion of their wives, and 4 cases showed infertility. In terms of semen parameters, 5 cases showed normozoospermia, 2 cases showed oligozoospermia, and 1 case showed azoospermia (Table 2). Anton et al^[23]^ reported that a carrier with 46, XY, t(5;17) (q31;p13) showed normozoospermia and infertility. The specific reasons deserve further exploration. These cases suggest that the fertility problems of RCT carriers with involvement of chromosome band 17p13 are complex.

To clarify the relationship between chromosome band 17p13 and phenotypes, GO analysis was performed. The results of molecular functions suggested that these targets genes were mostly involved in microfilament motor activity, ATPase activity. The results of biological processes suggested that these targets genes are involved in muscle contract, muscle filament slicing, skeleton muscle contract, ATP metabolic process, et cetera (Fig. 2). For case 3, oligoasthenospermia may be related to ATPase activity. These target genes involved in ATP metabolic process play an important role.

In addition, the short arm of chromosome 17 is very small, so it is difficult to be diagnosed through karyotype analysis, especially for chromosome 17p13.3. Alvaradon et al^[24]^ reported a patient with Miller-Dieker syndrome inherited from his father and he carried a balanced cryptic translocation with 46, XY, t(17;19) (p13.3q13.33). Hence, more molecular cytogenetic technology is required. Recently, some new molecular genetic techniques have been used to diagnose RCT, such as genome sequencing,^[15]^ fluorescence in situ hybridization,^[24]^ long-read sequencing,^[25]^ nanopore sequencing, and breakpoint region analysis.^[26]^

In summary, the fertility problems of RCT carriers are complex, and genetic counseling is still a challenge. More detailed cases should be collected to clarify the relationship between translocation breakpoint, related genes and clinical phenotype. The translocation chromosome and breakpoint analysis should be considered in the clinical assessment of the patients. Molecular genetic technology and bioinformatics analysis may provide more useful information for genetic counseling.

A limitation of this study is that molecular genetic analysis was not performed. We reviewed the fertility problems of male carriers involved in 17p13 translocation, which provides a basis for further genetic counseling.

## 5. Conclusion

In conclusion, we report 3 male carriers of RCT involving chromosome band 17p13, and review 14 reported cases of the same karyotype. Although chromosome 17 is closely related to spermatogenic failure, the seminal parameters of RCT carriers with 17p13 breakpoint are varied. Physicians should be aware of these breakpoints in genetic counseling. These breakpoints and the function of related genes require further study.

## Acknowledgments

We thank Ellen Knapp, PhD, from Liwen Bianji (Edanz) (www.liwenbianji.cn/), for editing the English text of a draft of this manuscript.

## Author contributions

**Conceptualization:** Ranwei Li.

**Data curation:** Ranwei Li.

**Investigation:** Ranwei Li.

**Writing** – **original draft:** Ranwei Li.

**Writing** – **review & editing:** Ranwei Li.
